# The mixed model for repeated measures for cluster randomized trials: a simulation study investigating bias and type I error with missing continuous data

**DOI:** 10.1186/s13063-020-4114-9

**Published:** 2020-02-07

**Authors:** Melanie L. Bell, Brooke A. Rabe

**Affiliations:** Department of Epidemiology and Biostatistics, Mel and Enid Zuckerman College of Public Health, University of Arizona, 1295 N Martin Ave, Tucson, AZ 85724 USA

**Keywords:** Missing data, Dropout, Variance components, Intention-to-treat, Cluster trials, Group randomized trials

## Abstract

**Background:**

Cluster randomized trials (CRTs) are a design used to test interventions where individual randomization is not appropriate. The mixed model for repeated measures (MMRM) is a popular choice for individually randomized trials with longitudinal continuous outcomes. This model’s appeal is due to avoidance of model misspecification and its unbiasedness for data missing completely at random or at random.

**Methods:**

We extended the MMRM to cluster randomized trials by adding a random intercept for the cluster and undertook a simulation experiment to investigate statistical properties when data are missing at random. We simulated cluster randomized trial data where the outcome was continuous and measured at baseline and three post-intervention time points. We varied the number of clusters, the cluster size, the intra-cluster correlation, missingness and the data-generation models. We demonstrate the MMRM-CRT with an example of a cluster randomized trial on cardiovascular disease prevention among diabetics.

**Results:**

When simulating a treatment effect at the final time point we found that estimates were unbiased when data were complete and when data were missing at random. Variance components were also largely unbiased. When simulating under the null, we found that type I error was largely nominal, although for a few specific cases it was as high as 0.081.

**Conclusions:**

Although there have been assertions that this model is inappropriate when there are more than two repeated measures on subjects, we found evidence to the contrary. We conclude that the MMRM for CRTs is a good analytic choice for cluster randomized trials with a continuous outcome measured longitudinally.

**Trial registration:**

ClinicalTrials.gov, ID: NCT02804698.

## Introduction

Cluster randomized trials (CRTs) are a design that randomizes clusters, rather than individuals, to intervention arms. The design may be used because the intervention is at the cluster level, such as behavioral group therapy, or due to potential contamination between participants, or because of ethical or logistic considerations [1]. Clusters may be households, clinics, schools or towns, and individuals within clusters are usually correlated, thereby violating the independence assumption of common statistical methods. The intracluster correlation (ICC), defined as the ratio of the between-cluster variance to the total variance, is the measure of this non-independence [1]. CRTs are increasingly being used as they are a good design for comparative effectiveness research and pragmatic trials [2].

Many trials, both individually and cluster randomized, have repeated outcome measures over time. For example, a cardiovascular disease (CVD) prevention trial may measure weight, body mass index and stress at baseline, at 3 months post intervention and a year [3]. The longitudinal design must be accounted for in the analysis because repeated measures on the same individual are not independent. Maximum-likelihood-based mixed models are one common statistical approach for handling non-independence. One particular type of mixed model, commonly referred to as the mixed model for repeated measures (MMRM), is a popular choice for individually randomized trials with longitudinal continuous outcomes measured at set time points [4–7]. This model uses an unstructured time and covariance structure and its appeal is due to (1) avoidance of model misspecification and (2) its unbiasedness for data which are missing completely at random (MCAR) or missing at random (MAR). Although many researchers use simple methods, such as a t-test to compare arms at a given time point, or single imputation such as last-observation-carried-forward [8–10]. these approaches can result in biased estimates; *t* tests make an MCAR assumption (defined below) and last-observation-carried-forward has been shown to give unpredictable results [11–13]. In addition to unbiased estimation when data are MAR or MCAR, mixed models are more powerful than *t* tests when data are missing [14].

Data are MCAR if missingness is unrelated to any observed or unobserved data (covariates or outcomes). Data are MAR if missingness is related to observed outcome data (such as the previous weight, in the CVD example above), but not unobserved data, such as the subject’s current weight. Data are said to be missing not at random (MNAR) if the missingness is dependent on unobserved data (such as the current weight measurement in the CVD example), even after taking observed data into account. A fourth category is sometimes used, which is covariate-dependent missing data. (Note that some researchers have called this type of missing data MCAR [15], whereas others have called it MAR [7, 16]) Statistical methods that result in unbiased estimation when data are MAR or MCAR include mixed models, multiple imputation, and inverse probability weighted generalized estimating equations. Reviews show that most trialists use methods that make the strong assumption that data are MCAR (e.g., *t* tests on available data, single imputation) [8–10]. A more conservative approach is to use a primary analysis that assumes that data are MAR, followed by a sensitivity analysis that weakens this assumption [11, 12].

CRTS can be analyzed at the individual or the cluster level, where data from each of the clusters is summarized by a single value, such as the mean (thereby removing the issue of intra-cluster correlation) [1]. For a small number of clusters (< 40 total) the recommendation is to use a cluster-level analysis [17]. particularly if unweighted generalized estimating equations are used, as type I error can be severely inflated otherwise [18]. With respect to missing data, Hossain et al. compared individual-level analysis versus cluster-level analysis for CRTs with covariate-dependent missing data where the continuous outcomes were measured twice (baseline and follow-up) [19]. They found that using mixed models or multiple imputation at the individual level resulted in unbiased estimation in all considered scenarios, whereas analysis at the cluster level did not always result in unbiased estimates. The focus of this paper is on analysis at the individual level, which is how most CRTs are analyzed [9, 17].

When the CRT has outcomes measured longitudinally on the same individual, both types of non-independence must be accounted for. Research into the most appropriate analytical approach for this type of design has been limited, particularly with respect to missing data. Johnson et al. [20] investigated type I error for several analytical approaches at the individual and cluster level for CRTs with imbalanced cluster size, but did not consider outcomes that were measured longitudinally. They recommended using the Kenward-Rogers denominator degrees of freedom, a small sample correction that has been shown to have favorable properties [21, 22]. Murray et al. investigated analytical approaches for CRTs with longitudinally measured outcomes, and concluded that mixed-model analyses of variance (ANOVAs) are inappropriate when there are more than two time points [23]. However, their conclusions may be too broad, as they did not test the model that we propose here.

We extend the MMRM to cluster trials (MMRM-CRT) by simply adding a random effect for cluster. While this model is not necessarily new; for example, Littell discusses this model in the context of repeated measures with clustering due to schools [24], the choice of similar models has been criticized when outcomes are measured at more than two time points (as mentioned above) [23, 25, 26]. Furthermore, to our knowledge, this model has not been investigated for its statistical properties when outcome data are incomplete. The objective of this research was to extend the MMRM to CRTs with continuous outcomes measured longitudinally on the same subject at more than two fixed time points, and to investigate this model’s statistical properties using simulation, particularly with respect to missing data. Specifically, the aims of this study were to investigate the bias of treatment effects and variance estimates, as well as the type I error rate of the MMRM-CRT. We aimed to investigate the impact of varying the ICC; the number of clusters per arm; cluster size; missingness mechanism; and underlying covariance structure. We demonstrate using a CRT for CVD prevention among diabetics, where the clusters are clinics from the state of Sonora, Mexico.

## Methods

### The MMRM in general

The mixed model for repeated measures uses an unstructured time and covariance structure [27]. Unstructured time means that time is modeled categorically, rather than continuously as a linear or polynomial function, and allows for an arbitrary trajectory over time. While the continuous time models may use fewer degrees of freedom and may, therefore, be more powerful, it can be difficult to anticipate the outcome’s time trajectory in advance. Since clinical trials often require a pre-specified analysis plan, unstructured time can be appealing [27]. In the context of randomized controlled trials, fixed effects of time, treatment and their interaction are included in the MMRM model. The interaction term accommodates different patterns of change over time between the arms. Baseline values of the outcome are sometimes included [28]. Maximum-likelihood-based mixed models provide unbiased estimation for data that are MCAR or MAR, as long as the model is not misspecified [29, 30]. All outcome data are used, regardless of whether an individual has complete data or not, making these models consistent with an intention-to-treat analysis [31–33].

Cluster randomized trials with longitudinally measured outcomes have two sources of non-independence: the cluster and the repeated measures over time. Linear mixed-effects models are one option for handling the non-independence of measurements over time. In the mixed-model context, one may use a random-coefficients model, using random effects for a subject’s intercept and sometimes slope. Alternatively, one may use covariance pattern models, where the covariance between repeated measures on the same subject is modeled explicitly from the residual effects [28, 30]. Some commonly used covariance structures, available in statistical software, include compound symmetric, autoregressive, Toeplitz or unstructured. A compound symmetric structure assumes that any two measurements on the same individual have the same covariance, regardless of timing. An autoregressive structure assumes that measurements’ correlation drops over time exponentially. The Toeplitz structure has homogeneous variance over time, and a banded correlation structure, so that the (r,c) element of the matrix is the same as the (r + 1, c + 1) and the (r + 2, c + 2) elements, etc. (i.e., when the degree of adjacency is the same) [28]. Unstructured covariance makes no assumptions about the correlation between measurements, thereby rendering misspecification not a problem; however, it can require that a large number of parameters must be estimated [30]. However, many cluster trials have a fairly small number of assessments on each subject.

The general mixed model for the ith subject is given by:

$$ {\displaystyle \begin{array}{c}{\mathrm{Y}}_{\mathrm{i}}={\mathrm{X}}_{\mathrm{i}}\upbeta +{\mathrm{Z}}_{\mathrm{i}}{\upgamma}_{\mathrm{i}}+{\upvarepsilon}_{\mathrm{i}}\\ {}{\upgamma}_{\mathrm{i}}\sim \mathrm{N}\left(0,\mathrm{G}\right)\\ {}{\upvarepsilon}_{\mathrm{i}}\sim \mathrm{N}\left(0,{\mathrm{R}}_{\mathrm{i}}\right)\end{array}} $$

where γ_i_ is independent of ε_i_; Y_i_ is the n_i_ × 1 response vector; n is the number of planned assessments for each subject i = 1, …, N and n_i_ is the number of observed assessments for the *i*th subject; β is the p × 1 fixed-effects vector; X_i_ is the n_i_ × p fixed-effects design matrix; Z_i_ is the n_i_ × q matrix of random-effects design matrix; γ_i_ is the q × 1 vector of random effects and ε_i_ is the n_i_ × 1 vector of residuals. G is the q × q covariance matrix for the random effects, and R_i_ is the n_i_ × n_i_ covariance matrix for the residuals.

### The MMRM for CRTs

Our proposed model, for a two-arm trial, has *p* = 2*n* + 2 fixed effects: a fixed effect for each assessment for each treatment arm, an intercept and a treatment indicator. The only random effect is for cluster, so q = 1, and G is a scalar. R_i_ is unstructured. This model is easily extended to include more than two arms, the baseline value of the outcome variable as a covariate (instead of in the outcome vector as shown here), and/or a baseline by treatment arm interaction [28].

### The ICC

The ICC is defined as $\frac{\sigma_C^2}{\sigma_{Total}^2}$ where between-cluster variance is ${\sigma}_C^2$ and ${\sigma}_{Total}^2$ is the total variance. In the MMRM-CRT model, ${\sigma}_{Total}^2$ is ${\sigma}_C^2+{\sigma}_B^2+{\sigma}_W^2$, where ${\sigma}_W^2$ is the within-subject (or residual) variance and ${\sigma}_B^2$ is the between-subject variance. These variance components are functions of the elements of the G and R matrices. The ICC is used to calculate the design effect = 1 + (*m* − 1) × ICC, where *m* = cluster size, which is the factor used to inflate the required sample size of an individually randomized trial to account for clustering, while maintaining the same level of power.

### Simulation study

We undertook a set of simulation studies to investigate the MMRM for CRTs in the presence of missing data. We simulated data from a two-arm CRT where the outcome was continuous and measured at baseline and three post-intervention time points. We varied the ICC, the number of clusters per arm *k*, the cluster size *m*, missingness (complete or MAR), and the missingness mechanism direction (described below). To show the generality of the MMRM-CRT we simulated data using three methods, which created different underlying covariance structures. The values for the simulation to investigate bias are shown in Table 1. We also simulated under the null hypothesis to investigate the type I error rate. We used 1000 replications for each parameter combination. We simulated MAR data only: MCAR data were not simulated because analyses which are unbiased for MAR data are also unbiased for MCAR data. Methods for MNAR data are beyond the scope of this paper.

**Table 1:** Simulation method 1^a^. Estimates (percent bias) of the difference at the fourth time point and variance components, where *k* = number of clusters per arm and *m* = cluster size

*k*	*m*	ICC = 0.01				ICC = 0.1			
		effect = 5	${\sigma}_c^2=1$	${\sigma}_w^2=39$	${\sigma}_b^2=60$	effect = 5	${\sigma}_c^2=10$	${\sigma}_w^2=30$	${\sigma}_b^2=60$
5	10	4.97 (− 0.7)	2.2 (120.8)	39.2 (0.4)	58.5 (− 2.5)	5.07 (1.5)	9.9 (− 0.8)	30.0 (0.0)	59.4 (− 0.9)
	20	4.94 (− 1.2)	1.4 (35.6)	39.1 (0.2)	59.6 (− 0.6)	5.04 (0.8)	10.4 (3.5)	30.0 (0.0)	60.0 (0.0)
	50	4.96 (− 0.8)	1.0 (4.4)	38.9 (− 0.2)	59.9 (− 0.2)	4.93 (−1.3)	10.3 (2.9)	30.1 (0.2)	60.2 (0.3)
10	10	5.02 (0.4)	1.8 (75.0)	39.0 (0.0)	59.3 (−1.2)	4.95 (− 1.0)	10.2 (1.7)	30.0 (0.0)	60.3 (0.5)
	20	5.01 (0.3)	1.2 (24.0)	39.0 (− 0.1)	59.7 (− 0.5)	4.98 (− 0.3)	9.8 (− 1.7)	30.0 (0.1)	59.8 (− 0.3)
	50	5.01 (0.1)	1.1 (5.0)	39.0 (0.1)	59.8 (− 0.3)	4.97 (− 0.6)	10.3 (2.8)	30.0 (0.0)	60.1 (0.1)
20	10	5.02 (0.5)	1.4 (36.9)	38.9 (− 0.2)	59.5 (− 0.9)	5.04 (0.8)	10.2 (1.7)	30.0 (0.0)	59.8 (− 0.4)
	20	5.01 (0.3)	1.1 (5.1)	39.0 (0.1)	59.9 (− 0.1)	5.02 (0.3)	10.0 (− 0.4)	30.0 (0.0)	60.1 (0.1)
	50	5.01 (0.1)	1.0 (3.3)	39.0 (0.0)	59.9 (− 0.2)	4.96 (− 0.7)	10.1 (0.8)	30.0 (0.0)	60.0 (0.0)
50	10	5.04 (0.7)	1.1 (13.6)	39.0 (0.0)	59.8 (− 0.4)	4.97 (− 0.6)	10.0 (0.5)	30.0 (0.0)	59.9 (− 0.1)
	20	4.98 (− 0.3)	1.0 (3.4)	39.0 (− 0.1)	60.0 (0.0)	5.06 (1.1)	10.0 (0.0)	30.0 (− 0.1)	59.9 (− 0.1)
	50	5.00 (− 0.1)	1.0 (− 0.8)	39.0 (0.0)	60.0 (0.1)	5.01 (0.2)	10.0 (− 0.1)	30.0 (0.0)	60.0 (0.0)

^a^Random effects for subject and cluster ≥ compound symmetric

*ICC* intracluster correlation

### Data generation

Multivariate normal data were generated using three methods, none of which are based directly on the model that we are proposing. To investigate bias, the means of *y* over time were (50, 50, 50, 50) for the control arm and (50, 55, 60, 55) for the treatment arm. To investigate type I error, we simulated under the null, with no differences in trajectories over time between the arms. Each method simulated data with ICCs of 0.01 and 0.1, values that are consistent with empirically estimated ICCs [34].

The first simulation method was a mixed-effects model with fixed effects for categorical time, treatment arm, and their interaction; random effects (intercepts) for subject and cluster; and a single residual-variance component, σ^2^_w_. The number of random effects is *q* = 2, so G is a 2 × 2 matrix comprised οϕ σ^2^_Χ_ and σ^2^_B_. This model induces a compound symmetric covariance structure for measurements on the same subject. The correlation for subjects within the same cluster is the ICC. For ICC = 0.01, we set σ^2^_C_=1, σ^2^_B_ = 60, and σ^2^_w_ = 39. For ICC = 0.1, we set σ^2^_C_ = 10, σ^2^_B_ = 60, and σ^2^_w_ = 30.

In the second simulation method we used the same fixed-effects structure as method 1, but with a single random cluster effect and a within-subject covariance over time governed by the following Toeplitz covariance matrix. For ICC = 0.01, we set σ^2^_C_ = 1 and σ^2^_w_ = 99. For ICC = 0.1, we set σ^2^_C_ = 10 and σ^2^_w_ = 90.

$$ R={\sigma}_W^2\left[\begin{array}{cccc}1& .8& .7& .6\\ {}.8& 1& .8& .7\\ {}.7& .8& 1& .8\\ {}.6& .7& .8& 1\end{array}\right] $$

The third data-simulation method used the same fixed-effects structure as the previous two methods, but included random effects for subject, cluster intercepts and cluster slopes. Each cluster *k* had a random effect generated ~ N(0, σ^2^_C_), which represents a constant offset from the overall mean trajectory over time, and additional random effects at each of the four time points ~ N(0, 0.4σ^2^_C_). he number of random effects is q = 5, so G is a 5 × 5 matrix. For ICC = 0.01,we set σ^2^_C_ = 0.714, σ^2^_B_ = 60, and σ^2^_w_ = 39. For ICC = 0.1, we set σ^2^_C_ = 7.14, σ^2^_B_ = 60, and σ^2^_w_ = 30. The simulation code is given in the Additional file 1.

### Missingness

We created MAR dropout data by using thresholds, above or below which data were deleted with a given probability at each post-baseline time point. The probability and thresholds were tuned so that data were missing at a rate of 30% in both arms at the final time point. Missingness at the jth time point was based on the outcome value of the j−1th time point, which creates MAR data. All baseline data were complete, and missingness was dropout only. Two mechanisms were used: “same direction,” where values of lower *y* were deleted for both arms; and “opposite direction,” where high values of *y* were more likely to be deleted in the control arm, and low values were more likely to be deleted in the treatment arm. In practice, missingness is likely to be a result of a combination of mechanisms, and we use this mechanism as an extreme case. However, a possible scenario where this type of missingness might occur is when the toxicity for the experimental arm is high so that patients with low quality of life are likely to drop out, whereas the control arm’s toxicity is low, and patients with higher quality of life might be likely to drop out, as they feel better, or possibly cured.

### Analysis

We analyzed each of the datasets using the MMRM-CRT as described above using the SAS Mixed procedure (version 9.4, Cary, NC, USA) with a random intercept for cluster, and fixed effects of categorical time, treatment, and the interaction time x treatment. The unstructured covariance is indicated within the repeated statement. The SAS and R code to fit this model is given in the Additional file 1. Our interest was focused on the difference at the fourth time point between the treatment arms, which we estimated using a contrast within the model. We used restricted maximum-likelihood estimation, and the Kenward-Rogers denominator degrees of freedom [22].

### Performance evaluation

The percent bias was calculated for the mean difference between the arms at the fourth time point by subtracting the true difference from the estimated difference and dividing by the true difference. Coverage was calculated as the percentage of 95% confidence intervals that contained the true value. We assessed the type I error rate for data that were simulated under the null hypothesis of no effect, by finding the percentage of tests (between arms at the fourth time point) that were significant at the 0.05 level. As bias was our performance measure of greatest interest, we calculated the Monte Carlo standard error for percent bias as:

$$ 100\times \left[\sqrt{Var(estimate)/{n}_{sim}}\right]/5, $$

where 5 was the true treatment difference [35].

We also estimated the bias of variance components. The estimates of variance generated by SAS are σ^2^_c_ from the random statement, and the 4 × 4 R matrix from the repeated statement, 10 elements of which are unique. Thus, the bias for the variance of the random effect of cluster, σ^2^_c_, was estimated directly, as this variance estimate is default output for SAS Proc Mixed. For data that were simulated using methods 1 and 3, the expected value of the average of the diagonal elements of R is σ^2^_B_ + σ^2^_W_ and the average of the off-diagonal elements should be equal to σ^2^_W_.

For data that were generated using method 2 (a Toeplitz covariance structure and a random effect for cluster), we calculated bias by dividing each of the elements of the estimated R matrix by its presumed correlation due to the Toeplitz structure, and averaged these values to get an estimate of σ^2^_W_. Diagonal elements were divided by 1, one place off the diagonal were divided by 0.8, two places off the diagonal were divided by 0.7, and three places off the diagonal were divided by 0.6.

## Simulation results

The direction of missingness did not affect the results, so we report results when missingness was in the same direction for both arms. Results for when missingness direction differed between arms are given in the Additional file 1.

### Bias of treatment effect

The estimates of the difference between arms at the fourth time point were largely unbiased (Tables 1, 2 and 3). The true treatment effect was 5.0: estimates range from 4.93 to 5.12 and the percent bias ranged from − 1.3 to 2.4%. There was no effect of the number of clusters, the cluster size, the ICC, the simulation method or the direction of missing data. The Monte Carlo standard error for the percent bias of the treatment effect ranged from 0.2 to 1.9%.

**Table 2:** Simulation method 2^a^. Estimates (percent bias) of effect (difference at the fourth time point) and variance components, where *k* = number of clusters per arm and *m* = cluster size

*k*	*m*	ICC = 0.01			ICC = 0.1		
		effect = 5	${\sigma}_c^2=1$	${\sigma}_w^2=99$	effect = 5	${\sigma}_c^2=10$	${\sigma}_w^2=90$
5	10	4.94 (− 1.2)	2.4 (137.2)	97.4 (− 1.6)	5.02 (0.5)	10.5 (4.5)	90.4 (0.4)
	20	4.94 (− 1.1)	1.6 (58.5)	98.8 (− 0.2)	5.09 (1.7)	9.7 (− 2.7)	89.8 (− 0.2)
	50	5.01 (0.3)	1.1 (8.6)	99.0 (0.0)	4.95 (− 0.9)	9.7 (− 2.8)	89.7 (− 0.4)
10	10	5.01 (0.3)	1.9 (85.6)	98.1 (− 0.9)	4.94 (− 1.2)	9.8 (− 2.0)	89.6 (− 0.4)
	20	5.03 (0.5)	1.3 (30.1)	98.6 (− 0.4)	4.99 (− 0.1)	10.0 (0.2)	89.8 (− 0.2)
	50	5.02 (0.4)	1.0 (4.8)	98.9 (− 0.1)	4.98 (− 0.5)	9.9 (− 1.0)	90.0 (0.0)
20	10	5.05 (1.1)	1.4 (41.4)	98.6 (− 0.4)	4.98 (− 0.4)	10.0 (− 0.2)	90.2 (0.2)
	20	4.97 (− 0.5)	1.1 (7.1)	98.6 (− 0.4)	4.96 (− 0.8)	10.1 (0.6)	90.1 (0.1)
	50	5.00 (0.0)	1.0 (− 0.8)	99.0 (0.0)	4.99 (− 0.2)	10.0 (0.5)	90.1 (0.1)
50	10	5.02 (0.4)	1.2 (20.2)	99.1 (0.1)	5.00 (0.0)	10.0 (0.4)	90.0 (0.0)
	20	5.02 (0.4)	1.0 (1.5)	98.9 (− 0.1)	5.02 (0.4)	10.0 (0.3)	90.0 (0.0)
	50	5.00 (0.0)	1.0 (3.0)	98.9 (− 0.1)	5.03 (0.5)	10.0 (0.1)	90.0 (0.0)

^a^Toeplitz covariance, random cluster effect

*ICC* intracluster correlation

**Table 3:** Simulation method 3^a^_._. Estimates (percent bias) of effect (difference at the fourth time point) and variance components, where *k* = number of clusters per arm and *m* = cluster size

*k*	*m*	ICC = 0.01				ICC = 0.1			
		effect = 5	${\sigma}_c^2=0.71$	${\sigma}_w^2=39$	${\sigma}_b^2=60$	effect = 5	${\sigma}_c^2=7.14$	${\sigma}_w^2=30$	${\sigma}_b^2=60$
5	10	5.03 (0.6)	2.0 (186.0)	39.6 (1.6)	58.9 (− 1.8)	4.99 (− 0.1)	8.2 (15.1)	32.4 (7.9)	59.7 (− 0.5)
	20	5.00 (0.1)	1.3 (76.6)	39.3 (0.7)	59.4 (− 1.0)	4.97 (− 0.6)	7.8 (9.0)	32.2 (7.4)	59.7 (− 0.5)
	50	4.95 (− 1.1)	0.8 (18.0)	39.2 (0.5)	60.0 (− 0.1)	4.97 (− 0.5)	8.0 (11.8)	32.3 (7.6)	59.5 (− 0.8)
10	10	5.03 (0.6)	1.7 (132.2)	39.2 (0.5)	59.1 (− 1.4)	4.98 (− 0.5)	8.0 (12.5)	32.7 (8.9)	58.9 (− 1.8)
	20	5.03 (0.5)	1.0 (43.7)	39.3 (0.7)	59.7 (− 0.5)	4.93 (− 1.4)	7.8 (9.2)	32.5 (8.4)	59.2 (− 1.4)
	50	4.96 (− 0.7)	0.8 (17.5)	39.3 (0.7)	59.7 (− 0.4)	5.12 (2.4)	7.8 (8.9)	32.5 (8.4)	59.3 (− 1.1)
20	10	4.98 (− 0.4)	1.2 (72.9)	39.3 (0.8)	59.5 (− 0.8)	5.04 (0.8)	7.8 (9.7)	32.8 (9.2)	59.5 (− 0.8)
	20	5.00 (0.0)	0.9 (26.5)	39.3 (0.8)	59.9 (− 0.2)	5.03 (0.6)	7.8 (9.2)	32.7 (9.0)	59.4 (− 1.1)
	50	5.01 (0.1)	0.8 (11.2)	39.3 (0.7)	60.0 (− 0.1)	4.95 (− 1.0)	7.9 (10.4)	32.7 (9.1)	59.4 (− 1.0)
50	10	5.00 (0.1)	0.9 (28.1)	39.3 (0.8)	59.7 (− 0.6)	4.98 (− 0.5)	7.8 (9.8)	32.8 (9.2)	59.3 (− 1.1)
	20	4.98 (− 0.5)	0.8 (10.1)	39.2 (0.6)	59.9 (− 0.1)	5.02 (0.4)	7.9 (10.5)	32.8 (9.4)	59.4 (− 1.1)

^a^Random effect for subject, cluster, and cluster slope

*ICC* intracluster correlation

### Bias of variance components

In general, variance component estimates were also unbiased: of the 192 variance components estimated, 85% had less than 10% bias. Smaller cluster sizes, particularly when the number of clusters was small, and low ICCs were associated with higher relative bias for σ^2^_C_. For example, when *k* = 5, *m* = 10 and ICC = 0.01, simulation methods 1–3 had percent biases of 121, 137, and 186%, respectively. The estimates for σ^2^_C_ were 2.2, 2.4, and 2.0 for true values of 1.0, 1.0, and 0.71. Using simulation method 3 estimates of the variance for cluster effects and within subject were slightly inflated and estimates for variance between subjects were slightly underestimated.

### Coverage

When the ICC was small, at 0.01, coverage estimates for the treatment effect were close to 95% for all three simulation methods. There was noticeable under-coverage when the ICC was 0.1 under simulation method 3 (random slope effect for cluster) with coverage falling as low as 89.7%. See Table 4.

**Table 4:** Coverage values for treatment effect (difference at the fourth time point) with 30% missing data in the same direction for each of the three simulation methods^a^

*k*, clusters per arm	*m*, subjects per cluster	Method 1		Method 2		Method 3	
		ICC = 0.01	ICC = 0.1	ICC = 0.01	ICC = 0.1	ICC = 0.01	ICC = 0.1
5	10	95.1	92.8	95.2	93.3	95.6	94.0
	20	92.1	93.3	94.4	92.9	95.3	92.0
	50	94.2	91.5	94.4	92.6	95.6	93.3
10	10	95.1	93.6	95.7	94.8	95.6	93.5
	20	94.1	94.8	95.6	94.6	95.3	90.7
	50	94.2	93.2	94.0	94.5	94.8	91.4
20	10	94.8	95.9	94.0	94.3	95.9	91.8
	20	94.4	94.2	94.6	93.8	94.4	92.9
	50	94.1	94.1	94.8	92.9	93.1	89.7
50	10	94.8	94.4	94.7	95.5	94.1	91.6
	20	94.2	94.5	94.8	95.3	95.1	90.3
	50	94.2	93.2	95.9	93.4	95.0	92.9

^a^Simulation method 1 = compound symmetry; method 2 = Toeplitz; method 3 = random intercepts and slopes

*ICC* intracluster correlation

### Type I error

When simulating under the null with 30% missing data, type I error ranged from 2.7 to 8.1% (Table 5). Larger values occurred using simulation method 3 (random intercept and slope for clusters) with larger ICC. Other methods and ICCs yielded values that were close to nominal.

**Table 5:** Type I error rate when estimating under the null hypothesis of no difference between arms, with 30% missing data in same direction for each of the three simulation methods^a^

*k*, clusters per arm	*m*, subjects per cluster	Method 1		Method 2		Method 3	
		ICC = 0.01	ICC = 0.1	ICC = 0.01	ICC = 0.1	ICC = 0.01	ICC = 0.1
5	10	3.9	6.3	2.7	4.4	4.9	6.5
	20	4.1	5.6	3.7	6.1	4.7	7.8
	50	5.2	4.4	5.4	4.1	4.8	6.8
10	10	4.1	6.0	4.8	5.6	5.0	5.6
	20	5.0	6.2	4.9	5.7	5.3	7.0
	50	4.7	4.2	5.3	5.4	6.4	8.1
20	10	5.0	4.3	5.3	4.1	4.3	6.4
	20	4.3	6.2	5.8	3.8	5.0	5.7
	50	4.8	5.2	4.8	4.3	5.6	5.7
50	10	5.0	5.8	4.8	4.9	6.5	6.5
	20	5.5	5.5	5.1	5.0	6.1	6.3
	50	5.2	4.7	4.5	5.1	6.4	6.7

^a^Simulation method 1 = compound symmetry; method 2 = Toeplitz; method 3 = random intercepts and slopes

#### Motivating example

We demonstrate the MMRM-CRT with Meta-Salud Diabetes, a CRT designed to reduce the risk of CVD in diabetics in the Mexican state of Sonora by focusing on improving healthy behaviors. Clusters were health clinics where the intervention was implemented, and randomization was stratified by region (north, south, central). Informed consent from all participants was given and ethical approval was obtained. Details can be found elsewhere [3]. Briefly, the primary outcome was the Framingham CVD risk score, which is a function of age, sex, blood pressure, cholesterol, smoking, and diabetic status, as detailed in D’Agostino et al. which estimates the risk of CVD in the next 10 years [36]. Twenty-four clinics were randomized to intervention (*n* = 293) or control (*n* = 242), with two clusters eventually being dropped from the control arm due to logistical reasons. Participants were assessed at baseline, 3, and 12 months. For this demonstration, we fit a MMRM-CRT with fixed effects of time, arm, time x arm, strata, and a random effect for clinics. Time was fit categorically and the 3 × 3 covariance matrix for time was unstructured. Inference focused on the difference in CVD risk between the arms at 3 and 12 months.

## Results

By month 12, the rates of missing outcome data were 21% and 11% for the intervention and control arm respectively. We found statistically significant differences in CVD risk at both 3 and 12 months. CVD risk was 4.8 percentage points higher in the intervention arm than the control arm at 3 months (95% CI 1.2, 8.5, *p* = 0.01); at 12 months the difference was 3.9 percentage points (95% CI 0.3, 7.4, *p* = 0.03). See Table 6. The ICC was estimated to be 0.031, similar to what other studies have found for various psycho-social and behavioral outcomes [34]. This trial had differential retention. While rates of missingness/retention should always be monitored and investigated by trial staff, unbiased estimation is still possible, as shown by Bell et al. [37]. In this particular case, it may have been due to the higher rate of participants who had just joined the health clinic in the intervention arm (34.1) versus the control arm (9.4), and were not fully committed to the clinic.

**Table 6:** Cardiovascular disease (CVD) risk from the Meta-Salud Diabetes cluster randomized trial at 3 and 12 months post intervention

Month	N_int_ missing (%)	N_ctl_ missing (%)	CVD risk (%) intervention *N* = 293	CVD risk (%) control *N* = 225	Difference (95% CI)	*p* value
0	1 (0.3)	6 (2.7)	21.0 (18.5, 23.6)	22.9 (20.1, 25.7)		
3	47 (16.0)	33 (14.7)	19.1 (16.6, 21.7)	24.0 (21.2, 26.8)	4.8 (1.2, 8.5)	0.01
12	61 (20.8)	31 (10.7)	19.3 (16.8, 21.9)	23.2 (20.5, 26.0)	3.9 (0.3, 7.4)	0.03

## Discussion

We aimed to investigate the mixed model for repeated measures for CRTs, for complete data and for data MAR, where assessments of the continuous outcome are made at fixed time points. When simulating a treatment effect at the final time point we found that estimates were unbiased when data were complete and when data were MAR. Estimates of variance components were mostly unbiased, although cluster effects were, in some cases, overestimated, particularly when the number of clusters per arm was small (*k* = 5) and when the data were simulated with random intercept and slopes for cluster. Although the percentage bias was large in some cases, up to 186%, this is due to a small true value of σ^2^_C_ = 0.71, and an average estimate of 2.0. In practice, this may not have much effect, but caution should be used whenever sample sizes are small, including when there are a small number of clusters with small cluster size.

Type I error rate was close to nominal for most of our simulation methods, ranging from 2.7 to 8.1% when simulating under the null. Generalized estimating equations, another popular approach for analyzing CRTs, also suffers from increased type I error when there is a small number of clusters. Huang et al. showed type I error of 47 to 12% when the number of clusters per arm ranged from two to five.

The worst performance for the MMRM-CRT occurred when the ICC was larger, at 0.1, and when simulation method 3 (random intercepts and slopes) was used. Empirical estimates of several ICCs in family practice settings had a median of 0.01; a similar study in the field of psycho-oncology had a median ICC of 0.0007 for longitudinal studies with a maximum value of 0.09 [34, 38]. This suggests that in certain research settings the ICC may be unlikely to be as high as 0.1. A way to reduce the ICC is to adjust for covariates within the models [34]. Real data are not generated from a model, and in practice, it is likely that multiple mechanisms are involved.

At the request of a reviewer, further simulations using non-linear trajectories for both arms were undertaken. The results are in the Additional file 1, and are similar to the main results: unbiased treatment estimation and variance components, except for slightly inflated between-cluster variance estimates when using simulation method 3, random intercepts and slopes. Type I error rate was similar to the primary results, with values slightly inflated for simulation method 3 and higher ICCs.

There have been multiple reviews that have asserted that the analyses for CRTs are incorrect based on whether a mixed-model ANOVA is used, if there are more than two time points, unless a random-

coefficients model is used [25, 26, 39]. Our results contradict this, as the MMRM-CRT appears, as a whole, to have good statistical properties. The simulation study upon which these assertions are based, however, did not test the MMRM-CRT as we have defined it, and was based on measurements on the same clusters over time, but not the same individuals over time [23]. Other differences include the low number of clusters simulated (five per arm); the assumption of a compound symmetric covariance structure, as opposed to the unstructured covariance in the MMRM-CRT; simulation under the null only; and the use of empirical sandwich standard errors as well as restricted maximum likelihood (REML). A low number of clusters, along with empirical sandwich errors, has been shown to increase type I error [18]. Hossain et al. recommend linear mixed models for the analysis of CRTs with missing data over cluster-level analyses, but their simulation study only used two time points.

While our study focused on endpoint analysis, i.e., comparison of arms at a single time point by using a contrast, the MMRM, for both individual and cluster randomization, can also assess response profiles over time. This allows for testing the difference in patterns of change over time between arms via the interaction of treatment and time [33].

### Strengths and limitations

A strength of this study is that we used three different data-generation models, none of which were directly the analysis model, as well as two mechanisms within these simulation methods (same and opposite direction missingness). Our results were fairly consistent, indicating that the MMRM-CRT is flexible and general. A limitation of this research is that, as a simulation study, we could not investigate all possible scenarios, of which there are an infinite number. However, this is a limitation of all simulation studies, and we varied several parameters that are important in practice. We only used three post-baseline time points; however, we see no reason why more time points would yield substantively different results. We did not simulate data that were MNAR. Although some studies have shown that MNAR data are modeled fairly well (in terms of bias) using MAR methods such as mixed models and multiple imputation, this is not true in general [31]. Another limitation is that we did not explore the case of unequal-sized clusters.

Most trials, both individual and cluster randomized, use analyses that make the strong and unlikely assumption that data are MCAR [8–10]. The MMRM makes a MAR assumption, which is more plausible than MCAR. While it is possible that longitudinal trial data are MNAR, MNAR models can be complex and most require strong untestable assumptions. We recommend that MNAR models be considered for sensitivity analyses. MNAR models for CRTs, particularly ones with repeated measures on the same subject, are an emerging research topic; for example, Fiero et al. extended the MNAR pattern-mixture model to longitudinal cluster trials [40]. Future research should include more MNAR models for CRTs, as well as analytical approaches for longitudinal binary and ordinal outcomes.

## Conclusion

The MMRM for individually randomized trials is popular because it uses all the data collected over time; is unlikely to misspecify the functional relationship between time and the outcome; and yields unbiased estimates for data that are MCAR or MAR. Our extension to cluster trials has similar properties, and can be considered as a primary analysis when continuous outcome data are collected at fixed time points.

## Supplementary information

**Additional file 1.** Mixed model for repeated measures-cluster randomized trials (Bell, Rabe).

## Data Availability

The simulation data can be recreated using the code from the online data files. The example data are proprietary and cannot be shared.

## Abbreviations

CRT: Cluster randomized trial
CVD: Cardiovascular disease
ICC: Intracluster correlation
MAR: Missing at random
MCAR: Missing completely at random
MMRM: Mixed model for repeated measures
MNAR: Missing not at random
