# Supplementary material for: The mixed model for repeated measures for cluster randomized trials: a simulation study investigating bias and type I error with missing continuous data
Source: Trials. 2020 Feb 7;21:148. doi: 10.1186/s13063-020-4114-9 (PMC7006144; doi:10.1186/s13063-020-4114-9)
Supplement: Supplementary file 1 — Additional file 1. Mixed model for repeated measures-cluster randomized trials (Bell, Rabe). [file 13063_2020_4114_MOESM1_ESM.docx]

**Appendix, Mixed Model for Repeated Measures-Cluster Randomized Trials (Bell, Rabe)**

Contents:

Box 1. SAS and R code for the MMRM-CRT

Table 1a-1c. Simulation results for MAR “different direction” mechanism.

Table 2. Coverage values for main effect for MAR “different direction” mechanism.

Table 3. Type I error rate for MAR “different direction” mechanism.

Table 4a-c. Simulation results for MAR “same direction” with non-linear trajectories for both arms.

Table 5. Type I error rate for MAR “same direction” mechanism with non-linear trajectories for both arms.

Box 2. Simulation code

Box 1. SAS and R code for the mixed model for repeated measure for cluster randomized trials (MMRM-CRT) where there are two treatment arms and four time points. The difference between treatment arms at the fourth time point is estimated. Cluster_id uniquely identifies each cluster: values are the same for every subject within a cluster.

| SAS (SAS Institute, Cary North Carolina)  PROC MIXED DATA = dataset;  CLASS time cluster_id subject_id;  MODEL y = treat time treat*time/DDFM = KR SOLUTION;  RANDOM int/SUBJECT = cluster_id;  REPEATED time/SUBJECT = subject_id TYPE = UN;  ESTIMATE 'Diff at time 4' treat 1 treat*time 0 0 0 1/CL;  run; |
| --- |
| R (The R Project for Statistical Computing, https://www.r-project.org)  library(nlme)  library(contrast)  # “treat” and “time” are factors with levels (0,1) and (0,1,2,3) respectively.  Model1 <- lme(y ~ treat*time,  random = ~ 1 \| cluster_id,  weights = varIdent(form = ~ 1 \| time),  correlation = corSymm(form = ~ 1 \| cluster_id/subject_id),  data = dataset, control = lmeControl(maxIter=10000,msMaxIter = 10000))  summary(Model1)  # Note that this contrast does not use the Kenward-Roger correction for degrees of  # freedom which is not implemented in nlme.  contrast(Model1,list(time = '3', treat = '1'),list(time = '3', treat = '0')) |

Table 1a-c. Results for differing directions (high values of y were more likely to be missing in the control arm, and low values were more likely to be deleted in the treatment arm). Estimates (percent bias) of effect and variance components under simulation method 1 (a), method 2 (b), and method 3 (c) with missing data in opposite directions. K denotes the number of clusters per arm, m denotes the number of subjects per cluster. 1000 simulations.

Table 1a. Simulation Method 1, random effects for subject and cluster => compound symmetric.

|  |  | ICC = 0.01 | | | | ICC = 0.1 | | | |
| --- | --- | --- | --- | --- | --- | --- | --- | --- | --- |
| k | **M** | $\text{effect}\boldsymbol{=5}$ | $\boldsymbol{\sigma}_{\boldsymbol{c}}^{\boldsymbol{2}}\boldsymbol{=1}$ | $\boldsymbol{\sigma}_{\boldsymbol{w}}^{\boldsymbol{2}}\boldsymbol{=39}$ | $\boldsymbol{\sigma}_{\boldsymbol{b}}^{\boldsymbol{2}}\boldsymbol{=60}$ | $\text{effect}\boldsymbol{=5}$ | $\boldsymbol{\sigma}_{\boldsymbol{c}}^{\boldsymbol{2}}\boldsymbol{=10}$ | $\boldsymbol{\sigma}_{\boldsymbol{w}}^{\boldsymbol{2}}\boldsymbol{=30}$ | $\boldsymbol{\sigma}_{\boldsymbol{b}}^{\boldsymbol{2}}\boldsymbol{=60}$ |
| 5 | **10** | 4.95 (-1.1) | 2.1 (107.0) | 38.9 (-0.3) | 59.5 (-0.8) | 5.08 (1.6) | 10.1 (0.7) | 29.9 (-0.4) | 60.7 (1.1) |
|  | **20** | 4.92 (-1.7) | 1.5 (50.4) | 38.9 (-0.2) | 59.6 (-0.7) | 4.96 (-0.8) | 10.1 (1.3) | 30.0 (0.0) | 59.8 (-0.4) |
|  | **50** | 4.98 (-0.5) | 1.1 (8.9) | 38.9 (-0.2) | 60.1 (0.1) | 5.08 (1.6) | 9.9 (-1.4) | 29.9 (-0.2) | 59.8 (-0.3) |
| 10 | **10** | 5.03 (0.6) | 1.7 (69.9) | 38.9 (-0.2) | 59.6 (-0.7) | 4.94 (-1.2) | 9.9 (-1.2) | 30.0 (0.0) | 59.9 (-0.2) |
|  | **20** | 5.00 (0.1) | 1.2 (24.2) | 39.0 (0.0) | 60.0 (-0.1) | 4.99 (-0.3) | 9.9 (-0.9) | 30.0 (0.1) | 59.7 (-0.5) |
|  | **50** | 5.01 (0.2) | 1.0 (-1.9) | 39.0 (0.0) | 59.9 (-0.1) | 4.97 (-0.6) | 10.0 (-0.5) | 30.0 (0.0) | 60.1 (0.2) |
| 20 | **10** | 5.07 (1.4) | 1.4 (38.1) | 38.9 (-0.3) | 59.4 (-1.0) | 5.06 (1.1) | 10.0 (0.4) | 30.1 (0.3) | 59.8 (-0.3) |
|  | **20** | 5.02 (0.4) | 1.0 (2.4) | 39.0 (0.1) | 60.1 (0.2) | 4.98 (-0.5) | 9.9 (-0.8) | 30.0 (0.1) | 60.1 (0.1) |
|  | **50** | 5.01 (0.1) | 1.0 (-0.9) | 39.0 (0.0) | 60.1 (0.2) | 5.01 (0.2) | 10.0 (-0.2) | 30.0 (0.0) | 59.9 (-0.1) |
| 50 | **10** | 5.01 (0.3) | 1.1 (13.9) | 38.9 (-0.1) | 59.8 (-0.3) | 5.01 (0.1) | 9.9 (-0.8) | 30.1 (0.2) | 60.1 (0.1) |
|  | **20** | 5.01 (0.2) | 1.0 (1.2) | 39.0 (0.1) | 60.1 (0.1) | 4.99 (-0.2) | 10.1 (0.5) | 30.0 (0.0) | 60.0 (0.0) |
|  | **50** | 4.98 (-0.5) | 1.0 (-0.4) | 39.0 (0.0) | 60.0 (0.0) | 4.99 (-0.1) | 10.0 (-0.4) | 30.0 (0.0) | 60.0 (0.0) |

Table 1b. Simulation method 2, Toeplitz

|  |  | ICC = 0.01 | | | ICC = 0.1 | | |
| --- | --- | --- | --- | --- | --- | --- | --- |
| k | **M** | $\text{effect}\boldsymbol{=5}$ | $\boldsymbol{\sigma}_{\boldsymbol{c}}^{\boldsymbol{2}}\boldsymbol{=1}$ | $\boldsymbol{\sigma}_{\boldsymbol{w}}^{\boldsymbol{2}}\boldsymbol{=99}$ | $\text{effect}\boldsymbol{=5}$ | $\boldsymbol{\sigma}_{\boldsymbol{c}}^{\boldsymbol{2}}\boldsymbol{=10}$ | $\boldsymbol{\sigma}_{\boldsymbol{w}}^{\boldsymbol{2}}\boldsymbol{=90}$ |
| 5 | **10** | 5.09 (1.8) | 2.6 (155.3) | 97.5 (-1.5) | 4.94 (-1.2) | 9.9 (-0.8) | 90.0 (0.0) |
|  | **20** | 5.05 (1.1) | 1.5 (48.4) | 98.2 (-0.8) | 5.00 (0.1) | 9.9 (-1.1) | 90.3 (0.3) |
|  | **50** | 5.04 (0.7) | 1.1 (5.9) | 99.1 (0.1) | 4.94 (-1.3) | 9.9 (-0.5) | 89.6 (-0.5) |
| 10 | **10** | 5.01 (0.1) | 1.7 (74.6) | 97.6 (-1.4) | 4.97 (-0.6) | 10.1 (0.8) | 90.4 (0.4) |
|  | **20** | 4.96 (-0.8) | 1.3 (27.0) | 98.5 (-0.5) | 5.01 (0.2) | 10.0 (0.0) | 89.8 (-0.2) |
|  | **50** | 4.99 (-0.2) | 1.1 (5.1) | 98.8 (-0.2) | 5.03 (0.5) | 9.8 (-2.0) | 89.8 (-0.2) |
| 20 | **10** | 5.02 (0.3) | 1.5 (52.5) | 98.4 (-0.6) | 5.06 (1.3) | 10.0 (-0.2) | 89.7 (-0.3) |
|  | **20** | 5.01 (0.2) | 1.1 (11.3) | 98.7 (-0.3) | 5.05 (1.0) | 10.1 (0.8) | 90.3 (0.3) |
|  | **50** | 5.02 (0.4) | 1.0 (0.2) | 99.0 (0.0) | 4.99 (-0.2) | 10.0 (0.0) | 89.9 (-0.1) |
| 50 | **10** | 4.98 (-0.4) | 1.1 (14.1) | 98.7 (-0.3) | 4.99 (-0.2) | 10.1 (1.1) | 89.8 (-0.3) |
|  | **20** | 4.99 (-0.3) | 1.0 (1.3) | 99.0 (0.0) | 4.94 (-1.3) | 10.1 (1.1) | 89.9 (-0.1) |
|  | **50** | 5.00 (-0.1) | 1.0 (-1.3) | 99.0 (0.0) | 4.96 (-0.8) | 10.0 (0.0) | 90.0 (0.0) |

Table 1c. Simulation method 3: Random intercepts and slopes*.*

|  |  | ICC = 0.01 | | | | ICC = 0.1 | | | |
| --- | --- | --- | --- | --- | --- | --- | --- | --- | --- |
| k | **m** | $\text{effect}\boldsymbol{=5}$ | $\boldsymbol{\sigma}_{\boldsymbol{c}}^{\boldsymbol{2}}\boldsymbol{=0.71}$ | $\boldsymbol{\sigma}_{\boldsymbol{w}}^{\boldsymbol{2}}\boldsymbol{=39}$ | $\boldsymbol{\sigma}_{\boldsymbol{b}}^{\boldsymbol{2}}\boldsymbol{=60}$ | $\text{effect}\boldsymbol{=5}$ | $\boldsymbol{\sigma}_{\boldsymbol{c}}^{\boldsymbol{2}}\boldsymbol{=7.14}$ | $\boldsymbol{\sigma}_{\boldsymbol{w}}^{\boldsymbol{2}}\boldsymbol{=30}$ | $\boldsymbol{\sigma}_{\boldsymbol{b}}^{\boldsymbol{2}}\boldsymbol{=60}$ |
| 5 | **10** | 4.95 (-1.1) | 2.0 (186.9) | 39.3 (0.7) | 58.7 (-2.2) | 4.95 (-0.9) | 7.7 (7.6) | 32.4 (8.1) | 59.4 (-1.0) |
|  | **20** | 4.98 (-0.5) | 1.3 (83.7) | 39.1 (0.4) | 59.2 (-1.3) | 4.96 (-0.8) | 7.9 (10.9) | 32.3 (7.6) | 59.4 (-1.0) |
|  | **50** | 5.03 (0.6) | 0.9 (31.5) | 39.2 (0.6) | 60.0 (0.1) | 4.90 (-2.0) | 8.0 (12.1) | 32.3 (7.6) | 59.5 (-0.8) |
| 10 | **10** | 4.97 (-0.6) | 1.5 (115.6) | 39.3 (0.7) | 59.3 (-1.2) | 4.99 (-0.2) | 7.9 (9.9) | 32.6 (8.8) | 59.6 (-0.7) |
|  | **20** | 4.95 (-0.9) | 1.0 (42.5) | 39.2 (0.6) | 59.8 (-0.4) | 5.14 (2.7) | 7.8 (9.9) | 32.6 (8.7) | 59.3 (-1.1) |
|  | **50** | 5.02 (0.5) | 0.8 (17.9) | 39.2 (0.6) | 59.9 (-0.2) | 5.01 (0.2) | 8.1 (13.0) | 32.5 (8.4) | 59.5 (-0.9) |
| 20 | **10** | 5.00 (0.0) | 1.1 (57.1) | 39.3 (0.8) | 59.8 (-0.3) | 4.93 (-1.4) | 7.9 (10.3) | 32.8 (9.3) | 59.4 (-1.0) |
|  | **20** | 4.98 (-0.3) | 0.9 (27.4) | 39.3 (0.8) | 59.8 (-0.3) | 4.93 (-1.3) | 7.7 (8.1) | 32.7 (9.1) | 59.5 (-0.8) |
|  | **50** | 5.06 (1.1) | 0.8 (9.0) | 39.3 (0.7) | 59.7 (-0.4) | 4.97 (-0.5) | 8.0 (11.4) | 32.7 (9.1) | 59.2 (-1.3) |
| 50 | **10** | 4.99 (-0.1) | 0.9 (28.0) | 39.3 (0.7) | 59.9 (-0.2) | 5.00 (-0.1) | 7.8 (9.8) | 32.8 (9.2) | 59.3 (-1.2) |
|  | **20** | 5.01 (0.1) | 0.8 (11.5) | 39.3 (0.7) | 59.9 (-0.1) | 4.97 (-0.7) | 7.9 (10.8) | 32.8 (9.5) | 59.3 (-1.1) |
|  | **50** | 4.98 (-0.3) | 0.8 (9.9) | 39.5 (1.2) | 60.0 (0.0) | 4.99 (-0.1) | 7.9 (10.4) | 32.6 (8.7) | 59.3 (-1.1) |

Table 2. Coverage values for main effect when missing data are in opposite directions. 1000 simulations.

|  | | Method 1* | | Method 2 | | Method 3 | |
| --- | --- | --- | --- | --- | --- | --- | --- |
| k, clusters  per arm | **m, subjects**  **per cluster** | **ICC=0.01** | **ICC=0.1** | **ICC=0.01** | **ICC=0.1** | **ICC=0.01** | **ICC=0.1** |
| 5 | **10** | 93.9 | 91.9 | 96.2 | 92.5 | 95.8 | 92.0 |
|  | **20** | 95.1 | 92.3 | 93.9 | 92.2 | 93.7 | 89.4 |
|  | **50** | 93.8 | 91.3 | 93.7 | 92.0 | 93.6 | 89.9 |
| 10 | **10** | 93.8 | 93.8 | 93.9 | 93.8 | 95.7 | 92.8 |
|  | **20** | 94.7 | 93.2 | 95.9 | 93.5 | 93.3 | 90.9 |
|  | **50** | 93.6 | 93.5 | 95.2 | 93.8 | 93.3 | 92.2 |
| 20 | **10** | 94.4 | 94.3 | 94.4 | 95.2 | 93.4 | 94.2 |
|  | **20** | 95.0 | 94.4 | 93.1 | 94.0 | 93.8 | 92.0 |
|  | **50** | 94.5 | 94.8 | 94.1 | 94.9 | 94.4 | 91.2 |
| 50 | **10** | 93.9 | 94.3 | 95.3 | 94.0 | 93.6 | 93.3 |
|  | **20** | 95.3 | 94.9 | 94.3 | 95.1 | 93.6 | 92.0 |
|  | **50** | 94.3 | 93.6 | 93.9 | 93.0 | 93.4 | 90.5 |

* Simulation method 1 = compound symmetry; method 2 = Toeplitz; method 3 = random intercepts and slopes

Table 3. Type I error rate when estimating under the null hypothesis of no difference between arms, with 30% missing data in opposite direction, for each of the three simulation methods.* 1000 simulations.

| Type I error rates | | | | | | | |
| --- | --- | --- | --- | --- | --- | --- | --- |
|  |  | **Method 1** | | **Method 2** | | **Method 3** | |
| k, clusters  per arm | **m, subjects**  **per cluster** | **ICC = 0.01** | **ICC = 0.1** | **ICC = 0.01** | **ICC = 0.1** | **ICC = 0.01** | **ICC = 0.1** |
| 5 | **10** | 3.7 | 4.4 | 5.2 | 5.1 | 5.7 | 6.4 |
|  | **20** | 3.9 | 5.8 | 3.4 | 3.9 | 4.1 | 6.2 |
|  | **50** | 4.9 | 5.1 | 3.9 | 3.9 | 4.8 | 7.5 |
| 10 | **10** | 5.1 | 5.6 | 4.9 | 5.2 | 4.3 | 5.9 |
|  | **20** | 5.5 | 5.8 | 5.1 | 5.2 | 5.5 | 5.9 |
|  | **50** | 6.5 | 4.8 | 5.5 | 3.8 | 6.2 | 9.2 |
| 20 | **10** | 4.0 | 5.3 | 4.7 | 3.5 | 5.6 | 6.1 |
|  | **20** | 5.7 | 5.0 | 5.1 | 5.3 | 6.2 | 6.4 |
|  | **50** | 5.4 | 5.8 | 6.2 | 5.5 | 6.8 | 7.3 |
| 50 | **10** | 4.6 | 5.3 | 4.8 | 3.9 | 5.9 | 7.5 |
|  | **20** | 5.5 | 6.6 | 4.7 | 4.0 | 6.0 | 6.4 |
|  | **50** | 5.2 | 4.9 | 4.7 | 6.3 | 6.0 | 8.3 |

* Simulation method 1 = compound symmetry; method 2 = Toeplitz; method 3 = random intercepts and slopes

Table 4a-c. Results for non-linear trajectories for both arms, MAR same direction missingness. Means were (50,55,55,50) for the control arm; (50, 55, 60, 55) for the treatment arm. Estimates (percent bias) of effect and variance components under simulation method 1 (a), method 2 (b), and method 3 (c) with 30% missing data. K denotes the number of clusters per arm, m denotes the number of subjects per cluster. 100 replications.

Table 4a. Simulation Method 1, random effects for subject and cluster => compound symmetric

|  |  | ICC = 0.01 | | | | ICC = 0.1 | | | |
| --- | --- | --- | --- | --- | --- | --- | --- | --- | --- |
| k | **M** | $\text{effect}\boldsymbol{=5}$ | $\boldsymbol{\sigma}_{\boldsymbol{c}}^{\boldsymbol{2}}\boldsymbol{=1}$ | $\boldsymbol{\sigma}_{\boldsymbol{w}}^{\boldsymbol{2}}\boldsymbol{=39}$ | $\boldsymbol{\sigma}_{\boldsymbol{b}}^{\boldsymbol{2}}\boldsymbol{=60}$ | $\text{effect}\boldsymbol{=5}$ | $\boldsymbol{\sigma}_{\boldsymbol{c}}^{\boldsymbol{2}}\boldsymbol{=10}$ | $\boldsymbol{\sigma}_{\boldsymbol{w}}^{\boldsymbol{2}}\boldsymbol{=30}$ | $\boldsymbol{\sigma}_{\boldsymbol{b}}^{\boldsymbol{2}}\boldsymbol{=60}$ |
| 5 | **10** | 5.44 (8.7) | 2.5 (148.0) | 38.4 (-1.6) | 58.5 (-2.5) | 4.78 (-4.3) | 10.1 (0.9) | 30.6 (2.0) | 60.3 (0.4) |
|  | **20** | 4.99 (-0.2) | 1.6 (59.0) | 37.5 (-3.9) | 59.0 (-1.6) | 5.63 (12.6) | 9.7 (2.8) | 30.2 (0.8) | 60.1 (-0.2) |
|  | **50** | 4.98 (-0.5) | 0.9 (-0.1) | 39.2 (0.5) | 60.0 (0.0) | 4.84 (-3.2) | 11.2 (12.0) | 30.3 (1.0) | 60.3 (-0.5) |
| 10 | **10** | 5.17 (3.4) | 1.3 (33.7) | 39.0 (0.0) | 59.7 (-0.5) | 5.13 (2.7) | 9.8 (-2.3) | 31.1 (3.8) | 61.0 (1.6) |
|  | **20** | 4.92 (-1.5) | 1.0 (0.2) | 39.2 (0.4) | 60.1 (0.3) | 4.76 (-4.8) | 10.5 (5.1) | 30.1 (0.5) | 60.1 (0.1) |
|  | **50** | 5.01 (0.3) | 1.0 (3.4) | 38.8 (-0.5) | 59.6 (-0.7) | 5.00 (0.0) | 9.5 (-5.1) | 30.3 (0.9) | 60.2 (0.4) |
| 20 | **10** | 4.98 (-0.3) | 1.5 (47.0) | 37.8 (-3.0) | 58.7 (-2.1) | 5.08 (1.6) | 9.4 (-6.1) | 29.8 (-0.3) | 59.7(-0.5) |
|  | **20** | 5.01 (0.2) | 1.0 (0.8) | 38.6 (-1.1) | 59.4 (-1.0) | 5.11 2.3) | 10.1 (0.9) | 31.0 (3.5) | 60.9 (1.4) |
|  | **50** | 5.01 (0.2 ) | 1.0 (4.1) | 39.0 (0.0) | 59.7 (-0.5) | 4.92 (-1.5) | 10.1 (0.2) | 30.3 (0.0) | 60.2(-0.3) |
| 50 | **10** | 5.08 (1.5) | 1.1 (12.8) | 39.0 (0.0) | 59.8 (-0.3) | 4.91 (-1.8) | 10.2 (2.2) | 30.2 (0.7) | 60.0 (0.0) |
|  | **20** | 5.02 (0.3) | 1.1 (13.3) | 39.1 (0.1) | 60.0 (0.0) | 4.98 (-0.4) | 9.7 (-2.5) | 30.3 (0.9) | 60.1 (0.2) |
|  | **50** | 4.95 (-1.0) | 1.0 (0.0) | 39.0 (0.0) | 59.8 (-0.3) | 5.08 (1.7) | 10.0 (0.0) | 30.0 (0.0) | 59.9 (-0.2) |

Table 4b. Simulation method 2, Toeplitz

|  |  | ICC = 0.01 | | | ICC = 0.1 | | |
| --- | --- | --- | --- | --- | --- | --- | --- |
| k | **M** | $\text{effect}\boldsymbol{=5}$ | $\boldsymbol{\sigma}_{\boldsymbol{c}}^{\boldsymbol{2}}\boldsymbol{=1}$ | $\boldsymbol{\sigma}_{\boldsymbol{w}}^{\boldsymbol{2}}\boldsymbol{=99}$ | $\text{effect}\boldsymbol{=5}$ | $\boldsymbol{\sigma}_{\boldsymbol{c}}^{\boldsymbol{2}}\boldsymbol{=10}$ | $\boldsymbol{\sigma}_{\boldsymbol{w}}^{\boldsymbol{2}}\boldsymbol{=90}$ |
| 5 | **10** | 5.01 (0.3) | 2.6 (164.2) | 97.4 (-1.6) | 5.55 (9.6) | 10.4 (0.8) | 87.6 (-2.7) |
|  | **20** | 5.07 (1.6) | 1.2 (23.5) | 98.6 (-0.5) | 5.01 (0.2) | 9.9 (-1.1) | 90.3 (0.3) |
|  | **50** | 4.97 (-0.6) | 1.0 (0.0) | 100.2 (1.3) | 5.10 (2.0) | 10.2 (2.1) | 89.8 (-0.2) |
| 10 | **10** | 5.00 (0.0) | 2.6 (159.5) | 97.7 (-1.3) | 4.72 (-5.6) | 10.4 (4.1) | 88.7 (-1.4) |
|  | **20** | 5.00 (0.0) | 1.3 (27.1) | 98.5 (-0.5) | 5.23 (5.8) | 9.7 (-2.6) | 90.1 (0.1) |
|  | **50** | 5.03 (0.7) | 1.1 (8.0) | 99.0 (0.0) | 4.84 (-3.3) | 9.9 (-0.5) | 89.8 (-0.2) |
| 20 | **10** | 5.00 (0.0) | 1.8 (84.5) | 99.0 (0.0) | 4.98 (-0.4) | 9.9 (-0.5) | 89.6 (-0.4) |
|  | **20** | 4.96 (-0.6) | 1.1 (4.1) | 97.6 (-0.3) | 5.11 (2.3) | 9.6 (-3.9) | 89.4 (-0.7) |
|  | **50** | 4.99 (-0.2) | 1.0 (0.2) | 98.4 (-1.4) | 4.93 (-1.4) | 9.9 (-1.4) | 89.2 (-0.9) |
| 50 | **10** | 4.98 (-0.2) | 1.3 (27.6) | 97.7 (-1.4) | 4.93 (-1.4) | 9.7 (-2.8) | 89.6 (-0.4) |
|  | **20** | 5.01 (0.3) | 1.0 (0.0) | 98.4 (-0.6) | 4.91 (-1.7) | 9.9 (-1.1) | 89.6 (-0.4) |
|  | **50** | 4.98 (-0.4) | 1.0 (0.0) | 99.5 (0.5) | 4.95 (-0.9) | 10.0 (0.0) | 90.5 (0.6) |

Table 4c. Simulation method 3: Random intercepts and slopes*.*

|  |  | ICC = 0.01 | | | | ICC = 0.1 | | | |
| --- | --- | --- | --- | --- | --- | --- | --- | --- | --- |
| k | **m** | $\text{effect}\boldsymbol{=5}$ | $\boldsymbol{\sigma}_{\boldsymbol{c}}^{\boldsymbol{2}}\boldsymbol{=0.71}$ | $\boldsymbol{\sigma}_{\boldsymbol{w}}^{\boldsymbol{2}}\boldsymbol{=39}$ | $\boldsymbol{\sigma}_{\boldsymbol{b}}^{\boldsymbol{2}}\boldsymbol{=60}$ | $\text{effect}\boldsymbol{=5}$ | $\boldsymbol{\sigma}_{\boldsymbol{c}}^{\boldsymbol{2}}\boldsymbol{=7.1}$ | $\boldsymbol{\sigma}_{\boldsymbol{w}}^{\boldsymbol{2}}\boldsymbol{=30}$ | $\boldsymbol{\sigma}_{\boldsymbol{b}}^{\boldsymbol{2}}\boldsymbol{=60}$ |
| 5 | **10** | 5.44 (8.8) | 2.2 (213.5) | 41.6 (6.7) | 61.8 (3.1) | 4.78 (-4.3) | 8.5 (9.6) | 32.9 (9.6) | 59.6 (-0.7) |
|  | **20** | 4.98 (-0.2) | 1.3 (81.6) | 41.0 (5.1) | 61.1 (1.9) | 5.63 (12.7) | 7.2 (8.5) | 32.6 (8.5) | 59.7 (-0.5) |
|  | **50** | 4.97 (-0.5) | 1.0 (35.8) | 38.8 (-0.4) | 59.3 (-1.2) | 4.84 (-3.2) | 8.0 (5.9) | 31.8 (5.9) | 59.2 (-1.3) |
| 10 | **10** | 5.17 (3.4) | 1.7 (139.9) | 38.3 (-1.7) | 58.6 (-2.4) | 5.13(2.7) | 7.9 (11.2) | 32.6 (8.7) | 59.8 (-0.7) |
|  | **20** | 4.92 (-1.5) | 1.1 (53.4) | 38.9 (-0.2) | 59.5 (-0.9) | 4.76 (-4.8) | 8.1 (13.6) | 33.1 (10.2) | 60.2 (0.4) |
|  | **50** | 5.01 (0.3) | 0.7 (-4.2) | 39.4 (1.1) | 60.1 (0.2) | 5.00 (0.06) | 7.7 (7.9) | 32.4 (8.1) | 59.7 (-0.6) |
| 20 | **10** | 4.98 (-0.3) | 1.2 (73.1) | 39.3 (0.9) | 59.8 (-0.3) | 5.01 (1.6) | 8.3 (15.8) | 31.6 (5.5) | 58.8 (-1.9) |
|  | **20** | 5.01(0.2) | 0.7 (0.9) | 39.3 (0.8) | 59.8 (-0.3) | 5.11 (2.3) | 7.9 (10.3) | 32.1 (6.9) | 59.2 (-1.4) |
|  | **50** | 5.07 (1.5) | 0.8 (18.1) | 39.1 (0.2) | 59.6 (-0.4) | 4.93 (1.5) | 8.0 (12.4) | 32.2 (7.4) | 59.3 (-1.1) |
| 50 | **10** | 5.02 (0.3) | 1.0 (35.5) | 38.9 (-0.2) | 59.8 (-0.4) | 4.91(-1.8) | 7.4 (3.9) | 32.9 (9.8) | 60.0 (0.0) |
|  | **20** | 4.95 (-1.0) | 0.7 (1.1) | 39.4 (1.0) | 59.9 (-0.2) | 4.98 (-0.4) | 7.8 (9.8) | 32.7 (9.1) | 59.7 (-0.4) |
|  | **50** | 5.07 (1.5) | 0.7 (3.0) | 39.5 (1.3) | 60.0 (0.0) | 5.08 (1.7) | 7.9 (11.2) | 32.4 (8.0) | 59.3 (-1.1) |

Table 5. Type I error rate when estimating under the null hypothesis of no difference between arms, with 30% missing data, for each of the three simulation methods.* Results for non-linear trajectories for both arms. Means were (50,50,55,55) for the control arm; (50, 55, 60, 55) for the treatment arm. 100 replications

| Type I error rates | | | | | | | |
| --- | --- | --- | --- | --- | --- | --- | --- |
|  |  | **Method 1** | | **Method 2** | | **Method 3** | |
| k, clusters  per arm | **m, subjects**  **per cluster** | **ICC = 0.01** | **ICC = 0.1** | **ICC = 0.01** | **ICC = 0.1** | **ICC = 0.01** | **ICC = 0.1** |
| 5 | **10** | 0 | 5 | 6 | 5 | 5 | 8 |
|  | **20** | 4 | 5 | 5 | 8 | 5 | 9 |
|  | **50** | 8 | 6 | 3 | 2 | 8 | 8 |
| 10 | **10** | 6 | 9 | 6 | 3 | 5 | 7 |
|  | **20** | 4 | 8 | 6 | 8 | 8 | 8 |
|  | **50** | 4 | 7 | 5 | 5 | 6 | 7 |
| 20 | **10** | 1 | 6 | 4 | 4 | 5 | 3 |
|  | **20** | 4 | 6 | 4 | 6 | 5 | 9 |
|  | **50** | 4 | 2 | 3 | 2 | 8 | 8 |
| 50 | **10** | 6 | 6 | 4 | 8 | 3 | 10 |
|  | **20** | 6 | 7 | 5 | 6 | 2 | 1 |
|  | **50** | 4 | 3 | 5 | 10 | 5 | 10 |

* Simulation method 1 = compound symmetry; method 2 = Toeplitz; method 3 = random intercepts and slopes

Box 2. Simulation code

| **************************************************************;  * Create MAR data, sim method 1 = compound symmetric correlation;  **************************************************************;  %macro sim1(num_rep, m,k, sig2c, sig2b, sig2w);  data complete;  do rep = 1 to &num_rep;  id = 0;  nn = &m*&k*2; k = &k;  do cluster = 1 to &k*2;  b_C = sqrt(&sig2c)*rannor(0);  do i = 1 to &m;  b_B = sqrt(&sig2b)*rannor(0);  do time = 0 to 3;  if time = 0 then id + 1;  if id<=nn/2 then treat = 0;  else treat = 1;  b_W = sqrt(&sig2W)*rannor(0);  sig2tot= &sig2c + &sig2B + &sig2W;  y = b_C + b_B + b_W + 50 + treat*(5*time);  if time = 3 and treat = 1 then y = y - 10;  icc = &sig2c/sig2tot;  output;  end;  end;  end;  end;  drop i nn b:;  run;  data parms; set complete (keep = icc); if _n_ = 1;  do rep = 1 to &num_rep;  m=&m; k=&k; sig2c=&sig2c; sig2b=&sig2b; sig2w=&sig2w;  output;  end;  run;  * create wide form;  proc transpose data=complete out=complete_wide prefix=y;  by rep treat cluster id ;  id time;  var y;  run;  * create MAR data using a threshholding mechanism;  * this gives 30% missing in each arm;  data MAR_wide; set complete_wide;  rannum = ranuni(0);  if treat = 1 then do;  if rannum > 0.50 and y1<55 then do;  y2 = .; y3 = .;  end;  if rannum > 0.50 and y2<60 then y3 = .;  end;  if treat = 0 then do;  if rannum > 0.55 and y1>52 then do;  y2 = .; y3 = .;  end;  if rannum > 0.45 and y2>52 then y3 = .;  end;  drop rannum;  run;  * back to long form;  proc transpose data = MAR_wide out = MAR;  by rep treat cluster id;  run;  data MAR; set MAR;  time=input(substr(_name_, 2), 5.);  nn = &m*&k*2;  if id<=nn/2 then treat = 0; else treat = 1;  drop _name_;  run;  **************************************************************;  * Analysis, MAR data, sim method 1;  **************************************************************;  proc mixed data = MAR;  class time cluster id;  model y = treat time treat*time/ddfm = kr s;  random int/subject = cluster ;  repeated time/subject = id type = un ;  estimate 't3' treat 1 treat*time 0 0 0 1/cl;  ods select estimates convergencestatus covparms;  ods output estimates = est convergencestatus = converge covparms = covparm;  by rep;  run;  data est;  merge parms est (keep = rep estimate stderr probt);  by rep; missing = "MAR";  run;  data converge;  merge parms converge;  by rep; missing = "MAR";  run;  data covparm;  merge parms covparm;  by rep; missing = "MAR";  run;  data lib.allest; set lib.allest est; run;  data lib.allconv; set lib.allconv converge; run;  data lib.allcovparm; set lib.allcovparm covparm; run;  %mend;  * initialize;  data lib.allest; if _n_ = 1 then delete; run;  data lib.allconv; if _n_ = 1 then delete; run;  data lib.allcovparm; if _n_ = 1 then delete; run;  %sim1(num_rep=1, k=10, m=10, sig2c=10, sig2b=60, sig2w=30);  * several other parameter combinations;  **************************************************************;  * Create MAR data, sim method 2, Toeplitz correlation structure;  **************************************************************;  data corr_trt(type=CORR) ;  input _TYPE_ $ 1-4 _NAME_ $ 9-10 y0 y1 y2 y3 ;  datalines ;  MEAN 50 55 60 55  STD . . . .  N 100 100 100 100  CORR Y0 1 .8 .7 .6  CORR Y1 .8 1 .8 .7  CORR Y2 .7 .8 1 .8  CORR Y3 .6 .7 .8 1  run;  data corr_ctl(type=CORR) ;  input _TYPE_ $ 1-4 _NAME_ $ 9-10 y0 y1 y2 y3 ;  datalines ;  MEAN 50 55 55 50  STD . . . .  N 100 100 100 100  CORR Y0 1 .8 .7 .6  CORR Y1 .8 1 .8 .7  CORR Y2 .7 .8 1 .8  CORR Y3 .6 .7 .8 1  run;  %macro sim2(num_rep, m,k, sig2c, sig2);  %let n_arm = %sysevalf(&k*&m);  * initialize;  data complete_wide; if _n_ = 1 then delete; run;  data corr_trt(type=CORR); set corr_trt;  if _type_ = "STD" then do; y0=sqrt(&sig2); y1=sqrt(&sig2); y2=sqrt(&sig2); y3=sqrt(&sig2); end;  data corr_ctl(type=CORR); set corr_ctl;  if _type_ = "STD" then do; y0=sqrt(&sig2);y1=sqrt(&sig2); y2=sqrt(&sig2); y3=sqrt(&sig2); end;  run;  %do rep = 1 %to &num_rep;  * create correlated data;  proc simnorm data=corr_trt outsim=wide_trt numreal = &n_arm seed = 0;  var y0-y3;  run;  proc simnorm data=corr_ctl outsim=wide_ctl numreal = &n_arm seed = 0;  var y0-y3;  run;  data wide_trt; set wide_trt(rename =(rnum = id)); treat=1; rep = &rep; cluster =1+ floor((id-1)/&m); run;  data wide_ctl; set wide_ctl; treat=0; rep = &rep; id = rnum + &n_arm; cluster =1+ floor((id-1)/&m); drop rnum; run;  proc sort data = wide_trt; by rep cluster id; run;  proc sort data = wide_ctl; by rep cluster id; run;  data wide; set wide_trt wide_ctl; run;  * get cluster level random effect;  data cluster;  rep = &rep;  do cluster = 1 to &k*2;  b_clust = sqrt(&sig2c)*rannor(0);  output;  end;  run;  data wide; merge cluster wide; by rep cluster;  y0=y0 + b_clust;  y1=y1 + b_clust;  y2=y2 + b_clust;  y3=y3 + b_clust;  output;  drop b_clust;  run;  data complete_wide; set complete_wide wide; run;  %end;  * get long form;  proc transpose data = complete_wide out = complete;  by rep cluster id treat;  run;  data complete; set complete;  time=input(substr(_name_, 2), 5.);  drop _name_;  rename col1 = y;  run;  data parms; set complete ; if _n_ = 1;  do rep = 1 to &num_rep;  m=&m; k=&k; sig2c=&sig2c; sig2=&sig2;  icc = sig2c/(sig2c+sig2);  output;  end;  drop y;  run;  * create MAR data using a threshholding mechanism;  * this gives 30% missing in each arm;  data MAR_wide; set complete_wide;  rannum = ranuni(0);  if treat = 1 then do;  if rannum > 0.55 and y1<54 then do;  y2 = .; y3 = .;  end;  if rannum > 0.60 and y2<55 then y3 = .;  end;  if treat = 0 then do;  if rannum > 0.55 and y1<51 then do;  y2 = .; y3 = .;  end;  if rannum > 0.65 and y2<55 then y3 = .;  end;  drop rannum;  run;  * back to long form;  proc transpose data = MAR_wide out = MAR;  by rep cluster id treat;  run;  data MAR; set MAR;  time=input(substr(_name_, 2), 5.);  drop _name_; rename col1 = y;  run;  **************************************************************;  * Analysis, MAR data, sim method 2;  **************************************************************;  proc mixed data = MAR;  class time cluster id;  model y = treat time treat*time/ddfm = kr s;  random int/subject = cluster ;  repeated time/subject = id type = un ;  estimate 't3' treat 1 treat*time 0 0 0 1/cl;  ods select estimates convergencestatus covparms;  ods output estimates = est convergencestatus = converge covparms = covparm;  by rep;  run;  data est;  merge parms est (keep = rep estimate stderr probt);  by rep; missing = "MAR";  run;  data converge;  merge parms converge;  by rep; missing = "MAR";  run;  data covparm;  merge parms covparm;  by rep; missing = "MAR";  run;  data lib2.allest; set lib2.allest est; run;  data lib2.allconv; set lib2.allconv converge; run;  data lib2.allcovparm; set lib2.allcovparm covparm; run;  %mend;  *initialize;  data lib2.allest; if _n_ = 1 then delete; run;  data lib2.allconv; if _n_ = 1 then delete; run;  data lib2.allcovparm; if _n_ = 1 then delete; run;  * icc = 0.01;  %sim2(num_rep=1000, k=5, m=10, sig2c=1, sig2=99);  * several other parm combinations;  **************************************************************;  * Create MAR data, sim method 3, random intercepts and slopes;  **************************************************************;  %macro sim3(num_rep, m,k, sig2c, sig2b, sig2w);  data complete;  do rep = 1 to &num_rep;  id = 0;  nn = &m*&k*2; k = &k;  do cluster = 1 to &k*2;  b_C = sqrt(&sig2c)*rannor(0); *random intercept for cluster;  b_Ct0 = sqrt(.4*&sig2c)*rannor(0); *random slope for cluster;  b_Ct1 = sqrt(.4*&sig2c)*rannor(0); *random slope for cluster;  b_Ct2 = sqrt(.4*&sig2c)*rannor(0); *random slope for cluster;  b_Ct3 = sqrt(.4*&sig2c)*rannor(0); *random slope for cluster;  do i = 1 to &m;  b_B = sqrt(&sig2b)*rannor(0);  do time = 0 to 3;  if time = 0 then id + 1;  if id<=nn/2 then treat = 0;  else treat = 1;  b_W = sqrt(&sig2W)*rannor(0);  sig2tot= &sig2c*1.4 + &sig2B + &sig2W ; *note that cluster slope is added in here;  y = b_C + b_B + b_W + 50 + treat*(5*time);  if time = 3 and treat = 1 then y = y - 10;  icc = &sig2c*1.4/sig2tot;  output;  end;  end;  end;  end;  drop i nn ;  run;  data complete; set complete;  if time = 0 then y = y + b_Ct0;  if time = 1 then y = y + b_Ct1;  if time = 2 then y = y + b_Ct2;  if time = 3 then y = y + b_Ct3;  run;  data parms; set complete (keep = icc); if _n_ = 1;  do rep = 1 to &num_rep;  m=&m; k=&k; sig2c=&sig2c; sig2b=&sig2b; sig2w=&sig2w;  output;  end;  run;  * create wide form;  proc transpose data=complete out=complete_wide prefix=y;  by rep treat cluster id ;  id time;  var y;  run;  * create MAR data using a threshholding mechanism;  * this gives 30% missing in each arm;  data MAR_wide; set complete_wide;  rannum = ranuni(0);  if treat = 1 then do;  if rannum > 0.52 and y1<55 then do;  y2 = .; y3 = .;  end;  if rannum > 0.52 and y2<60 then y3 = .;  end;  if treat = 0 then do;  if rannum > 0.65 and y1<55 then do;  y2 = .; y3 = .;  end;  if rannum > 0.65 and y2<60 then y3 = .;  end;  drop rannum;  run;  * back to long form;  proc transpose data = MAR_wide out = MAR;  by rep treat cluster id;  run;  data MAR; set MAR;  time=input(substr(_name_, 2), 5.);  nn = &m*&k*2;  if id<=nn/2 then treat = 0; else treat = 1;  drop _name_;  run;  **************************************************************/;  * Analysis, MAR data, sim method 3;  **************************************************************;  proc mixed data = MAR;  class time cluster id;  model y = treat time treat*time/ddfm = kr s;  random int/subject = cluster ;  repeated time/subject = id type = un ;  estimate 't3' treat 1 treat*time 0 0 0 1/cl;  ods select estimates convergencestatus covparms;  ods output estimates = est convergencestatus = converge covparms = covparm;  by rep;  run;  data est;  merge parms est (keep = rep estimate stderr probt);  by rep; missing = "MAR";  run;  data converge;  merge parms converge;  by rep; missing = "MAR";  run;  data covparm;  merge parms covparm;  by rep; missing = "MAR";  run;  data lib3.allest; set lib3.allest est; run;  data lib3.allconv; set lib3.allconv converge; run;  data lib3.allcovparm; set lib3.allcovparm covparm; run;  %mend;  * initialize;  data lib3.allest; if _n_ = 1 then delete; run;  data lib3.allconv; if _n_ = 1 then delete; run;  data lib3.allcovparm; if _n_ = 1 then delete; run;  * icc = 0.01;  %sim3(num_rep=1000, k=5, m=10, sig2c=1, sig2b=60, sig2w=39);  * several other parameter combinations; |
| --- |
